# *Porphyromonas gingivalis* Induces Apoptosis and Autophagy via ER Stress in Human Umbilical Vein Endothelial Cells

**DOI:** 10.1155/2018/1967506

**Published:** 2018-07-29

**Authors:** Masaaki Hirasawa, Tomoko Kurita-Ochiai

**Affiliations:** Department of Microbiology and Immunology, Nihon University School of Dentistry at Matsudo, Matsudo-shi, Chiba-ken, Japan

## Abstract

It has been reported that periodontitis is associated with an increased risk of atherosclerosis. Accumulating evidence suggests that endothelial dysfunction is an early marker for atherosclerosis. To determine how periodontal infections contribute to endothelial dysfunction, we examined the effect of *Porphyromonas gingivalis* on human umbilical vein endothelial cells (HUVEC). *P. gingivalis* significantly suppressed the viability of HUVEC, induced DNA fragmentation and annexin V staining, and increased caspase-3, caspase-8, and caspase-9 activities. *P. gingivalis* also increased the expression of GADD153 and GRP78 and caspase-12 activity. Further, *P. gingivalis* induced autophagy, as evidenced by increased LC3-II and Beclin-1 levels. The suppression of *P. gingivalis*-induced autophagy by silencing of LC3 with siRNA significantly increased *P. gingivalis*-induced apoptosis. ER stress inhibitor, salubrinal, suppressed apoptosis and autophagy by inhibiting *P. gingivalis*-induced DNA fragmentation and LC3-II expression. These data suggest that *P. gingivalis* infection induces ER stress-mediated apoptosis followed by autophagic response that protects HUVEC from *P. gingivalis*-mediated apoptosis, potentially amplifying proatherogenic mechanisms in the perturbed vasculature.

## 1. Introduction

Periodontal disease is highly prevalent, affecting up to 90% of the global population [1]. *Porphyromonas gingivalis*, a major periodontal pathogen, was recently implicated in the pathogenesis of atherosclerosis [2]. *P. gingivalis* can directly access the systemic circulation and the endothelium in patients with periodontitis, as transient bacteremias are common [3]. Indeed, *P. gingivalis* has been detected in human atherosclerotic plaques [4, 5], and it can both invade endothelial cells (EC) and persist therein [6]. Further, *P. gingivalis* elicits a proatherogenic response in EC in the form of increased leukocyte adhesion with concomitant upregulation of adhesion molecules and proinflammatory cytokines and chemokines [7, 8]. Interestingly, these effects require the invasion of EC by viable bacteria [8].

EC are key cellular components of blood vessels that function as a selectively permeable barrier between blood and tissue. It is believed that atherogenic risk factors induce apoptosis in EC, leading to the denudation or dysfunction of the intact endothelial monolayer, with subsequent atherosclerotic lesion formation as a result of lipid accumulation, monocyte adhesion, and inflammatory reactions [9, 10]. Endothelial cell apoptosis has several potential deleterious effects, including plaque erosion and thrombosis [11]. Recent studies have demonstrated increased endoplasmic reticulum (ER) stress protein expression in the vascular cells of atherosclerotic lesions and regulation of the protein in the endothelium by several atherosclerotic stressors [12].

Autophagy is a cellular defense mechanism involving degradation and recycling of cytoplasmic components. Autophagy can protect cells from apoptosis; thus, it is said to sit at the crossroad between cell death and survival. However, excessive autophagy can destroy essential cellular components and cause cell death [13]. Accumulating evidence has also suggested that ER stress is linked to autophagy [14]. In the present study, we examined the capacity of whole, viable *P. gingivalis* to induce cell death via apoptosis, ER stress, and autophagy in human umbilical vein endothelial cells (HUVEC).

## 2. Materials and Methods

### 2.1. Bacterial Strains and Culture Methods

*P. gingivalis* strain 381 and KDP136 (gingipain-null mutant) were cultured on anaerobic blood agar plates (Becton Dickinson Co., Sunnyvale, CA) in a model 1024 anaerobic system (Forma Scientific, Marietta, OH) under 10% H_2_, 80% N_2_, and 10% CO_2_ for 3–5 days. The cells were then inoculated into brain heart infusion broth (Difco Laboratories, Detroit, MI) supplemented with 5 *μ*g/ml hemin and 0.4 *μ*g/ml menadione and cultured to an OD_660_ of 0.8 (10^9^ cfu/ml). The cells were then harvested by centrifugation at 8000 ×g for 20 min at 4°C and diluted in phosphate-buffered saline (PBS).

### 2.2. Cell Line and Reagents

HUVEC (provided by Lonza-Takara, Tokyo, Japan) were cultured at 37°C in a humidified atmosphere with 5% CO_2_ in endothelial cell culture medium (EGM-2 BulletKit, Lonza-Takara). All experiments were performed on cells at passages 4–8 at approximately 80% confluence.

### 2.3. Cell Viability Assay and Cytotoxicity Assay

Cell viability was determined using a Cell Counting Kit-8 (CCK-8; Wako, Osaka, Japan). Briefly, cells (1.0 × 10^4^/well) were cultured in 100 *μ*l of endothelial cell culture medium in a 96-well plate and stimulated with *P. gingivalis* at the indicated multiplicity of infection (MOI). After 19 h, 10 *μ*l of CCK-8 solution was added, and the cells were incubated at 37°C for 2 h, followed by measurement of A450 using a spectrophotometer.

### 2.4. Measurement of Cell Death

Cellular apoptosis was quantified by DNA fragmentation using the Cell Death Detection ELISA^PLUS^ kit (Roche Diagnostics, Mannheim, Germany). Briefly, HUVEC (5 × 10^5^/dish) were cultured with *P. gingivalis* 381 and KDP136 strains at the indicated MOI. After 7, 16, and 24 h, the cells were lysed in 200 *μ*l of lysis buffer, and 20 *μ*l of the supernatant was reacted with 80 *μ*l of anti-DNA immunocomplex conjugated with peroxidase, which interacts with streptavidin-coated wells, in a microtiter plate for 2 h. At the end of the incubation, 100 *μ*l of substrate was added, and color development was quantified as a wavelength of 405 nm. The results were calculated as the ratio of the absorbance of the *P. gingivalis*-treated cells to the absorbance of the nontreated control cells. For the apoptosis inhibition assays, cells were preincubated for 1 h with caspase-12 inhibitor Z-VAD-FMK (MBL, Nagoya, Japan) or ER stress inhibitor, salbrinal (50 *μ*M) (Sigma-Aldrich, St. Louis, MO, USA), before stimulation with the bacteria. Apoptosis of the *P. gingivalis*-treated HUVEC was assessed by annexin V-EnzoGold and 7-amino-actinomycin D (7-AAD) staining (Enzo Life Sciences Inc., Farmingdale, NY) and flow cytometric analysis using a FACSCalibur Flow Cytometer (Becton Dickinson, Franklin Lakes, NJ).

### 2.5. Caspase Assay

After incubation (5 × 10^5^ cells/dish) for 16 h with *P. gingivalis* 381, the cells were harvested, and the caspase-1, caspase-3, caspase-8, caspase-9, and caspase-12 activities were measured using a caspase fluorometric protease assay kit (MBL). The amount of 7-amino-4-trifluoromethylcoumarine (AFC) released was measured using an ARVO multilabel/luminescence counter with excitation and emission at 400 and 505 nm, respectively.

### 2.6. Real-Time Quantitative RT-PCR

Quantitative RT-PCR was performed using primers specific for C/EBP homologous protein (CHOP)/growth arrest and DNA damage 153 (GADD153) (GGCAGCTGAGTCATTGCC and GCAGATTCACCATTCGGTCA), glucose-regulated protein-78 (GRP78) (CCTAGCTGTGTCAGAATCTCCATCC and GTTTCAATGTCACCATCCAAGATCC), Beclin-1 (CCAGATGCGTTATGCCCAGAC and CATTCCATTCCACGGGAACAC), microtubule-associated protein 1 light chain 3B (MAP1LC3B) (ACGCATTTGCCATCACAGTTG and GGGACCTTCAGCAGTTTACAGTCAG), and *β*-actin (CATCCGTAAAGACCTCTATGCCAAC and ATGGAGCCACCGATCCACA). The thermal cycling profile was as follows: 95°C for 10 s followed by 40 cycles of 95°C for 5 s and 60°C for 30 s, with a final dissociation at 95°C for 5 s, 60°C for 30 s, and 95°C for 15 s. The starting amount of RNA was quantified using a standard curve; fold changes in the expression of CHOP/GADD153, GRP78, Beclin-1, and LC3B relative to *β*-actin were determined in triplicate.

### 2.7. Antibodies and Western Blot Analysis

Rabbit antibodies against GADD153, GRP78, Beclin-1, and *β*-actin were purchased from Santa Cruz Biotechnology Inc. (Santa Cruz, CA), while those against LC-3 were purchased from MBL. Secondary horseradish peroxidase- (HRP-) conjugated goat anti-rabbit and goat anti-mouse antibodies were obtained from Amersham Pharmacia Biotech (Piscataway, NJ). Cells were lysed in a buffer containing 10 mM Tris-HCl, 150 mM NaCl, 1% Nonidet P-40, 1 mM ethylenediaminetetraacetic acid (EDTA), 1 mM ethylene glycol tetraacetic acid (EGTA), 0.1 mM phenylmethylsulfonyl fluoride, 8 *μ*g/ml aprotinin, and 2 *μ*g/ml leupeptin (pH 7.4). For immunoblotting, proteins resolved by 12.5% sodium dodecyl sulfate-polyacrylamide gel (SDS-PAGE) were transferred to polyvinylidene fluoride membranes (Millipore, Bedford, MA), which were then exposed to primary and then secondary antibodies. Chemiluminescence detection was performed with an ECL™ Western Blotting Detection Kit (Amersham). The signal intensities of the corresponding bands were measured by a Light Capture equipped with CS Analyzer software (ATTO, Osaka, Japan).

### 2.8. siRNA Knockdown

Knockdown of endogenous LC3 with siRNA was carried out using Lipofectamine RNAiMAX (Invitrogen) according to the manufacturer's instructions. Briefly, HUVEC grown to 50–80% confluence in a 6-well plate were transfected with either LC3B siRNA (sc-43391, Santa Cruz Biotechnology) or control siRNA (sc-37007, Santa Cruz Biotechnology). The final concentration of respective siRNA was 50 nM for each transfection, and the experiments were carried out 24 h after transfection.

### 2.9. Acridine Orange Staining

Cells were treated with *P. gingivalis* 381 at an MOI 1 : 10^2^ for 8 h and stained with 1 mg/ml acridine orange at room temperature for 20 min. Then, cells were washed with PBS and visualized by fluorescence microscopy.

### 2.10. Statistical Analysis

All data are presented as means ± SEM. Multiple-group comparisons were made by one-way analysis of variance, followed by post hoc intergroup comparison by the Bonferroni-Dunn test. A *p* value < 0.05 was considered statistically significant.

## 3. Results

### 3.1. *P. gingivalis* 381 Inhibits Cell Proliferation and Induces Cell Death through Apoptosis

**Figure 1:**
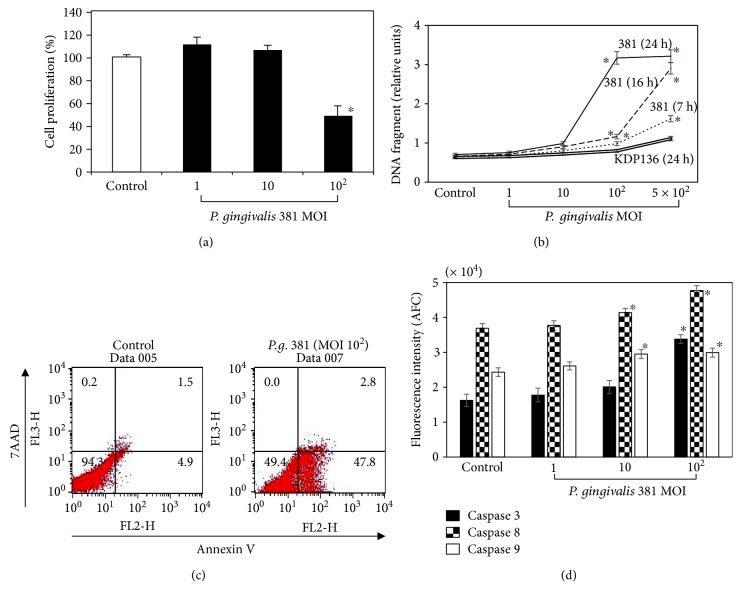
*P. gingivalis* infection inhibits cellular proliferation and induces apoptosis in HUVEC. (a) HUVEC were treated with *P. gingivalis* 381 at an MOI of 1 : 1–1 : 10^2^ for 21 h. Cell viability was determined using a Cell Counting Kit-8 (CCK-8). The data are expressed as the mean ± SEM of 3 different experiments. ^∗^*p* < 0.05 versus bacteria-free control cells. (b) HUVEC were treated with *P. gingivalis* 381 at an MOI of 1 : 1–1 : 5 × 10^2^ for 7, 16, and 24 h, and with KDP136 at an MOI of 1 : 1–1 : 5 × 10^2^ for 24 h. Cellular apoptosis was quantified by DNA fragmentation using the Cell Death Detection ELISA^PLUS^ Kit as described in Materials and Methods. Data are expressed as the mean ± SEM of 3 different experiments. ^∗^*p* < 0.05 versus bacteria-free control cells. (c) HUVEC were stained with annexin V-EnzoGold and 7-AAD after treatment with *P. gingivalis* 381 at an MOI of 1 : 10^2^ for 21 h, and then they were analyzed by flow cytometry. The figure is representative of three experiments with similar results. (d) HUVEC were treated with *P. gingivalis* 381 at an MOI of 1 : 1–1 : 10^2^ for 16 h. Cell extracts were prepared, and caspase activities were measured. Data are expressed as the mean ± SEM of 3 different experiments. ^∗^*p* < 0.05 versus bacteria-free control cells.

*P. gingivalis* 381 significantly diminished the proliferation of HUVEC at higher MOI (Figure 1(a)). Next, we investigated whether the suppression of cell proliferation observed in HUVEC treated with *P. gingivalis* 381 was dependent on apoptosis. Incubation with *P. gingivalis* 381 strongly induced apoptosis in the HUVEC in a dose- and time-dependent manner (Figure 1(b)). Higher apoptosis rates were observed at MOI of 1 : 5 × 10^2^ after 16 h and 1 : 10^2^ and 1 : 5 × 10^2^ after 24 h compared to the nontreated control group. In contrast, KDP 136 strain did not cause DNA fragmentation in any MOI and 24 h culture. In addition, we confirmed the possible apoptotic effect of *P. gingivalis* 381 by annexin V and 7-AAD staining and flow cytometric analysis (Figure 1(c)). The early apoptotic cells represented by the lower right quadrant (7-AAD negative and annexin V positive) and the nonviable necrotic and late-state apoptotic cells represented by the upper right quadrant (positive for annexin V binding and 7-AAD uptake). After 21 h of treatment with *P. gingivalis* 381 at an MOI of 1 : 10^2^, the number of early-stage apoptotic cells was significantly increased by up to 47.8 ± 2.0% (*p* < 0.01). These results indicated that the decrease in viable cells induced by *P. gingivalis* 381 treatment was secondary to apoptosis. Apoptotic cell death typically occurs via the stimulation of caspase activity [15]. Caspase-8 and caspase-9 appear to be activated in the death receptor- and mitochondrial-dependent apoptotic pathways, respectively, and caspase-3, which is common to both pathways, induces DNA fragmentation. Pyroptosis, which is uniquely dependent on caspase-1, has been described in monocytes, macrophages, and dendritic cells infected with a range of microbial pathogens such as *Salmonella*, *Francisella*, and *Legionella* [16]. Therefore, we assessed the activities of caspase-1, caspase-3, caspase-8, and caspase-9. *P. gingivalis* 381 enhanced caspase-3, caspase-8, and caspase-9 activities, suggesting involvement of the mitochondria-mediated intrinsic pathway and death receptor-induced extrinsic pathway (Figure 1(d)). On the other hand, caspase-1 activity was not enhanced by *P. gingivalis* 381 within the range of the MOI investigated (data not shown). Therefore, cell death induced by *P. gingivalis* 381 was unrelated to pyroptosis.

### 3.2. ER Stress Is Involved in *P. gingivalis* 381-Induced Apoptosis

**Figure 2:**
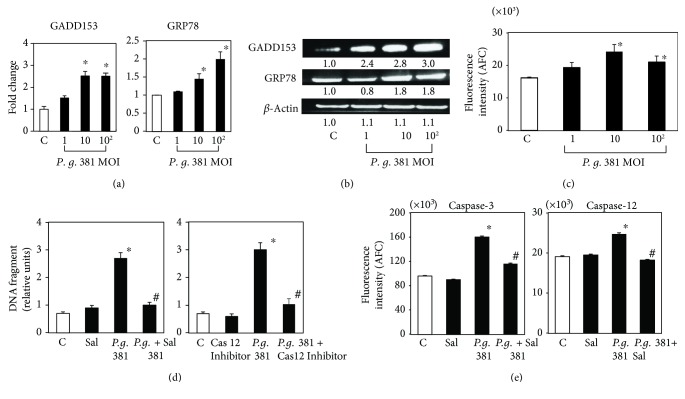
*P. gingivalis* 381 induces UPR-like response in HUVEC. (a) HUVEC were treated with *P. gingivalis* 381 at an MOI of 1 : 1–1 : 10^2^ for 8 h. Quantitative real-time PCR analysis was performed for GADD153 and GRP78 relative to *β*-actin expression. Data are expressed as the mean ± SEM of 3 different experiments. ^∗^*p* < 0.01 versus bacteria-free control cells. (b) HUVEC were treated with *P. gingivalis* 381 at an MOI of 1 : 1–1 : 10^2^ for 21 h. Whole cell lysates were subjected to SDS-PAGE, followed by Western blot analysis using antibodies to GADD153 and GRP78. Levels of *β*-actin were also detected as an internal control. The density of each band was measured by densitometry, and the relative band densities were calculated by comparing the band densities with those of the control. (c) HUVEC were treated with *P. gingivalis* 381 at an MOI of 1 : 1–1 : 10^2^ for 16 h. Cell extracts were then prepared, and caspase-12 activity was measured. Data are expressed as the mean ± SEM of 3 different experiments. ^∗^*p* < 0.01 versus bacteria-free control cells. (d) HUVEC were pretreated with a caspase-12 or ER stress inhibitor for 1 h and then treated with *P. gingivalis* 381 at an MOI of 1 : 10^2^ for 24 h. The ratio of apoptotic cells to total cells was determined using the Cell Death Detection ELISA^PLUS^ Kit. Data are expressed as the mean ± SEM of 3 different experiments. ^∗^*p* < 0.01 versus bacteria-free control cells. ^#^*p* < 0.01 versus bacteria-treated control cells. (e) HUVEC were pretreated with an ER stress inhibitor for 1 h and then treated with *P. gingivalis* 381 at an MOI of 1 : 10^2^ for 16 h. Cell extracts were then prepared, and caspase activity was measured. Data are expressed as the mean ± SEM of 3 different experiments. ^∗^*p* < 0.01 versus bacteria-free control cells. ^#^*p* < 0.01 versus bacteria-treated control cells.

To determine whether ER-mediated events contribute to *P. gingivalis* 381-induced apoptosis, we examined the levels of several substances reported to participate in ER-induced apoptosis and the unfolded protein response (UPR). Real-time PCR and Western blot analysis demonstrated that the ER stress-induced proteins or genes GADD153 and GRP78 were upregulated by treatment with *P. gingivalis* 381 (Figures 2(a) and 2(b)), although there was not so drastic but still significant difference in GRP78 mRNA level. A significant increase in caspase-12 activity at 21 h after the addition of *P. gingivalis* 381 was also observed at MOI of 1 : 10 and 1 : 10^2^ (Figure 2(c)). Furthermore, DNA fragmentation induced by *P. gingivalis* 381 infection was significantly suppressed by pretreatment with salubrinal and a caspase-12 inhibitor (Figure 2(d)). *P. gingivalis* 381-induced caspase-3 and caspase-12 activities were also completely abrogated by pretreatment with salubrinal (Figure 2(e)). These data suggest that the ER stress response functions prior to *P. gingivalis* 381-mediated apoptosis.

### 3.3. *P. gingivalis* 381 Contributes to ER Stress-Induced Autophagy

**Figure 3:**
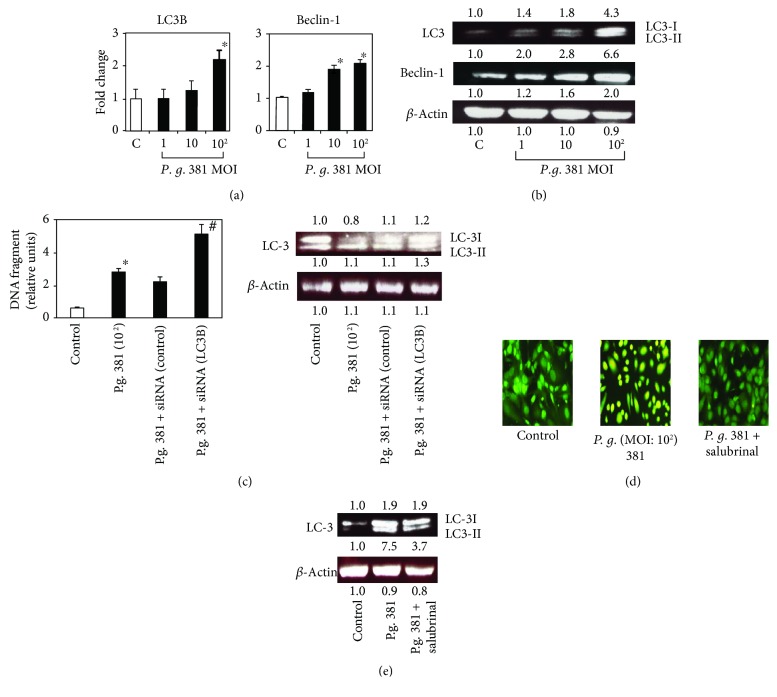
*P. gingivalis* 381 triggers ER stress-induced autophagy in HUVEC. (a) HUVEC were treated with *P. gingivalis* 381 at an MOI of 1 : 1–1 : 10^2^ for 8 h. Quantitative real-time PCR analysis was performed for LC3B and Beclin-1 relative to *β*-actin expression. Data are expressed as the mean ± SEM of 3 different experiments. ^∗^*p* < 0.01 versus bacteria-free control cells. (b) HUVEC were treated with *P. gingivalis* 381 at an MOI of 1 : 1–1 : 10^2^ for 21 h. Whole cell lysates were subjected to SDS-PAGE, followed by Western blot analysis using antibodies to LC3 and Beclin-1. Levels of *β*-actin were also detected as an internal control. The density of each band was measured by densitometry, and the relative band densities were calculated by comparing the band densities with those of the control. (c) HUVEC were transfected with 50 nM of LC3B-specific siRNA or control siRNA and then stimulated with *P. gingivalis* 381 at an MOI of 1 : 10^2^ for 8 h (real-time PCR) or 16 h (Western blotting). The ratio of apoptotic cells to total cells was determined using the Cell Death Detection ELISA^PLUS^ Kit. Data are expressed as the mean ± SEM of 3 different experiments. ^∗^*p* < 0.01 versus bacteria-free control cells. ^#^*p* < 0.01 versus nontransfected or control siRNA-treated cells. The expression of LC3 was analyzed by Western blotting. Lysates were then immunoblotted with anti-LC-3. Levels of *β*-actin were also detected as an internal control. (d) HUVEC were pretreated with an ER stress inhibitor for 1 h and then treated with *P. gingivalis* 381 at an MOI of 1 : 10^2^ for 8 h. Acridine orange staining indicated that the ER stress inhibitor suppressed autophagic vacuolation induced by *P. gingivalis* 381. (e) HUVEC were pretreated with an ER stress inhibitor for 1 h and then treated with *P. gingivalis* 381 at an MOI of 1 : 10^2^ for 21 h. Whole cell lysates were subjected to SDS-PAGE, followed by Western blot analysis using antibodies to LC3. Levels of *β*-actin were also detected as an internal control. The density of each band was measured by densitometry, and the relative band densities were calculated by comparing the band densities with those of the control.

Autophagy is essential for the removal of damaged organelles and long-lived cytosolic macromolecules to maintain energy homeostasis, and hence cell survival, under starvation conditions. When excessive, however, autophagy results in autophagic cell death. To examine the connection between *P. gingivalis* 381 infection and the stimulation of autophagy, we examined the expression of Atg6 (Beclin-1) and the colocalization of LC3-II (both are autophagosome markers). Real-time PCR and Western blot analysis demonstrated that LC3-II and Beclin-1 were upregulated by treatment with *P. gingivalis* 381 (Figures 3(a) and 3(b)). In order to examine if autophagic signaling contributes to the protection against cell death, autophagy (a ratio of LC3-II/LC3-I) was suppressed by siRNA specific for LC3 (Figure 3(c)). *P. gingivalis* 381 significantly increased apoptosis in autophagy-deficient HUVEC. These results suggest that autophagy induced by *P. gingivalis* 381 protects HUVEC from apoptosis caused by the same bacteria.

Autophagy is also characterized by acidic vesicular organelle (AVO) formation, which can be assayed by acridine orange staining [17]. AVOs accumulated in the cytoplasm of HUVEC exposed to *P. gingivalis* 381 (Figure 3(d)); however, this was inhibited by the addition of salubrinal (Figure 3(d)). Furthermore, salubrinal suppressed *P. gingivalis* 381-induced LC3-II expression (Figure 3(e)). These data suggest that the ER stress response functions prior to autophagy induced by *P. gingivalis* 381 infection.

## 4. Discussion

In this study, we found that HUVEC challenge with high doses of *P. gingivalis* 381 for 21 h significantly exhibited suppression of viability. And it was found that this decrease in viability is cell death caused by endothelial apoptosis as evidenced by DNA fragmentation, annexin V staining, and caspase activity (Figures 1(a) and 1(d)). Furthermore, since the challenge with KDP136 did not induce DNA fragmentation, it was suggested that the apoptosis-inducing factor in *P. gingivalis* 381 may be caused by gingipain (Figure 1(b)). Caspase-3, caspase-8, and caspase-9 were significantly activated by intermediate high doses of *P. gingivalis* 381 infection, suggesting that multiple signaling pathways were involved. Therefore, activation of these caspases may contribute to endothelial dysfunction. Pyroptosis is a more recently recognized form of regulated cell death with morphological and biochemical properties distinct from necrosis and apoptosis [16]. However, the death of the HUVEC in this study was unrelated to pyroptosis since caspase-1 activity was not changed at any *P. gingivalis* 381 dose examined (data not shown).

In this study, *P. gingivalis* 381 induced the expression of a number of ER stress markers, including GADD153, GRP78, and caspase-12, indicating that *P. ginigivalis* 381 induces ER stress. The mRNA and protein levels of GADD153 and GRP78 were increased in MOI concentration dependently except for the mRNA level of GADD153, but the caspase-12 activity was maximized at MOI (10) and decreased at MOI (10^2^). Because gingipain of *P. gingivalis* can cleave caspase-3 [18], cleavage of caspase-12 may be occurring at high concentration of MOI. Furthermore, since addition of either an ER stress inhibitor (salburinal) or an anti-caspase-12 reagent significantly decreased *P. gingivalis* 381-induced apoptosis in HUVEC, ER stress may occur upstream rather than downstream of apoptosis in cells exposed to high doses of *P. gingivalis* 381. Therefore, the activation of ER stress markers such as GADD153, GRP78, and caspase-12 will likely to happen at an early stage compared with the factors related to apoptosis. Induction of the mammalian UPR involves, in part, enhanced transcription of genes encoding ER chaperones, including BiP/GRP78, which serves to correct protein misfolding. The UPR, which serves to restore cellular homeostasis, induces the transcription of genes encoding antiapoptotic and proapoptotic proteins. Thus, severe or prolonged ER stress may induce apoptosis. Again, our data suggest that *P. gingivalis* 381 induces apoptosis after ER stress. ER stress has been demonstrated at all stages of atherosclerotic lesion development in ApoE knockout mice, and it is evident in human atherosclerotic lesions [12, 19, 20]. However, the relationship between *P. gingivalis* 381 infection and ER stress is yet to be determined. Our preliminary data indicated that the infection with a higher *P. gingivalis* 381 MOI induced reactive oxygen species (ROS) in HUVEC (data not shown). It is known that ROS induces the ER stress [21, 22]. Further, it was also found that lipopolysaccharide (LPS) could activate the ER stress [23]. Therefore, ROS and LPS release in HUVEC by infection with *P. gingivalis* might induce the ER stress to HUVEC. Indeed, the expression levels of UPR-related genes were significantly higher in periodontitis compared with gingivitis lesions [24].

Accumulating evidence suggests that the ER plays an essential role not only in apoptosis but also in the regulation of autophagy [14, 25]. However, whether ER stress-mediated autophagy contributes to cell survival or cell death remains unclear. Here, we found that *P. gingivalis* 381-induced ER stress enhanced autophagy. The process of autophagosome formation depends on several autophagy proteins [26]. By translational modification of LC3, LC3-II (16 kDa) localizes exclusively to autophagosomal membranes and has been used as an autophagy marker [27]. We demonstrated that *P. gingivalis* 381 induced the expression of autophagy markers (e.g., Beclin-1, LC3-II, and AVOs), and that LC3-II and AVO expressions were inhibited by salubrinal. In addition, since specific suppression of LC3 by siRNA effectively downregulated LC3-II/LC3-I ratio and significantly increased the apoptosis in HUVEC upon stimulation with *P. gingivalis* 381, the autophagy may protect HUVEC from *P. gingivalis* 381-induced apoptosis. Most likely, autophagy under basal conditions plays an important role in cellular housekeeping, whereas induced autophagy may function as a death pathway, as suggested by our results.

In summary, our data highlight the important relationships of ER stress and autophagy with apoptosis in HUVEC during *P. gingivalis* 381 exposure. Our data also provide putative mechanisms for *P. gingivalis* 381-induced endothelial dysfunction, as well as suggesting potential strategies for the prevention of *P. gingivalis* 381-induced endothelial impairment.

## 5. Conclusions

The experiments revealed that *P. gingivalis* 381 infection induces ER stress-mediated apoptosis followed by autophagic response that protects HUVEC from *P. gingivalis* 381-mediated apoptosis, potentially amplifying proatherogenic mechanisms in the perturbed vasculature.

## Data Availability

The data used to support the findings of this study are available from the corresponding author upon request.
